# Testing ontogenetic patterns of sexual size dimorphism against expectations of the expensive tissue hypothesis, an intraspecific example using oyster toadfish (*Opsanus tau*)

**DOI:** 10.1002/ece3.3835

**Published:** 2018-03-02

**Authors:** Alex Dornburg, Dan L. Warren, Katerina L. Zapfe, Richard Morris, Teresa L. Iglesias, April Lamb, Gabriela Hogue, Laura Lukas, Richard Wong

**Affiliations:** ^1^ North Carolina Museum of Natural Sciences Raleigh NC USA; ^2^ Senckenberg Institute for Biodiversity and Climate Frankfurt am Main Germany; ^3^ Physics and Biology Unit Okinawa Institute of Science and Technology Graduate University Okinawa Japan; ^4^ Department of Applied Ecology North Carolina State University Raleigh NC USA; ^5^ Delaware Division of Fish and Wildlife Dover DE USA

**Keywords:** evolutionary ecology, fishes, life history trade‐offs, phenotypic evolution, reproductive physiology, swim bladder

## Abstract

Trade‐offs associated with sexual size dimorphism (SSD) are well documented across the Tree of Life. However, studies of SSD often do not consider potential investment trade‐offs between metabolically expensive structures under sexual selection and other morphological modules. Based on the expectations of the expensive tissue hypothesis, investment in one metabolically expensive structure should come at the direct cost of investment in another. Here, we examine allometric trends in the ontogeny of oyster toadfish (*Opsanus tau*) to test whether investment in structures known to have been influenced by strong sexual selection conform to these expectations. Despite recovering clear changes in the ontogeny of a sexually selected trait between males and females, we find no evidence for predicted ontogenetic trade‐offs with metabolically expensive organs. Our results are part of a growing body of work demonstrating that increased investment in one structure does not necessarily drive a wholesale loss of mass in one or more organs.

## 1 INTRODUCTION

Pronounced differences in ecology, life history, or morphology between males and females of the same species are common features of the vertebrate Tree of Life (Barrett & Hough, 2012; Herler, Kerschbaumer, Mitteroecker, Postl, & Sturmbauer, 2010; Karp et al., 2017; Lamb et al., 2017; Nottebohm & Arnold, 1976). Sexual size dimorphism (SSD), the variation between sexes in aspects of size, is a particularly striking pattern that has commanded the attention of researchers since Darwin (Clutton‐Brock, Harvey, & Rudder, 1977; Darwin, 1871; Rohner, Blanckenhorn, & Puniamoorthy, 2016; Scudder, 1876; Shine, 1978). The past several decades have yielded remarkable insights into the eco‐evolutionary dynamics (Legrand & Morse, 2000; Maan & Seehausen, 2011; Price, 1984; Sonerud et al., 2012), evolutionary trade‐offs (Dunn et al., 2015; Gustafsson, Qvarnström, & Sheldon, 1995; Simmons & Emlen, 2006), and ontogeny underlying the rise of traits that exhibit SSD (German, 2004; Glassman, Coelho, Carey, & Bramblett, 1984; Hassell, Meyers, Billman, Rasmussen, & Belk, 2012; Holton, Alsamawi, Yokley, & Froehle, 2016). However, potential energetic trade‐offs between selection for SSD and investment in metabolically expensive organs are rarely considered, precluding a broader understanding of how SSD shapes fundamental aspects of phenotypic evolution in vertebrates.

The evolution of SSD requires selection to promote changes in some aspect of allometric growth (Bonduriansky, 2007). However, how modular these changes are remain unclear. Do such ontogenetic changes reflect trade‐offs with other components of a given species’ bauplan? This question is particularly relevant for SSD in metabolically or developmentally costly organs, as organisms are faced with a finite energy budget that they can invest into different structures in order to accumulate biomass. This raises the question of not only how organisms have evolved the sometimes extreme differences in organ size observed today, but also whether there are hidden costs to SSD. An often invoked answer to the generalized question of how organisms are able to change biomass investment in metabolically expensive organs was first conceptualized by Aiello and Wheeler (1995) in the form of the expensive tissue hypothesis (ETH). This hypothesis specifically posited that investment in a major metabolically expensive organ, the brain, should come at a cost to one or more other organ systems. As costly traits characterized by SSD (such as gonads or ornaments) become expressed, expectations of the ETH suggest that energy budgets will be differentially balanced between sexes, thereby driving reduced investment in the brain or other structures for the sex under selection. While the ubiquity of trade‐offs in life history evolution provides intuitive appeal for the ETH, evidence supporting the expectations of this hypothesis has not been overwhelming.

Interspecific studies of metabolic trade‐offs between organ systems have yielded mixed results for the ETH that include positive (Liao, Lou, Zeng, & Kotrschal, 2016; Sukhum, Freiler, Wang, & Carlson, 2016; Tsuboi et al., 2015), negative (Bordes, Morand, & Krasnov, 2011; Jones & MacLarnon, 2004), or a lack of support (Isler & van Schaik, 2006; Lemaître, Ramm, Barton, & Stockley, 2009; Navarrete, van Schaik, & Isler, 2011; Schillaci, 2006) for the ETH. Likewise, intraspecific studies have yielded a mixture of positive support (Kotrschal, Corral‐Lopez, Szidat, & Kolm, 2015; Kotrschal, Kolm, & Penn, 2016; Kotrschal et al., 2013) and inconclusive/negative evidence (Warren & Iglesias, 2012). It is important to consider that the ETH was initially formulated with the intent of understanding size variation in the vertebrate brain (Aiello & Wheeler, 1995), and therefore, work investigating trade‐offs has been almost entirely focused on the potential costs associated with increased brain size. Whether the ETH provides a predictive framework for understanding the impact of SSD in gonads or other costly organs in development remain unclear. Does SSD limit investment in the brain or other organs consistent with the expectations of the ETH?

**Figure 1:**
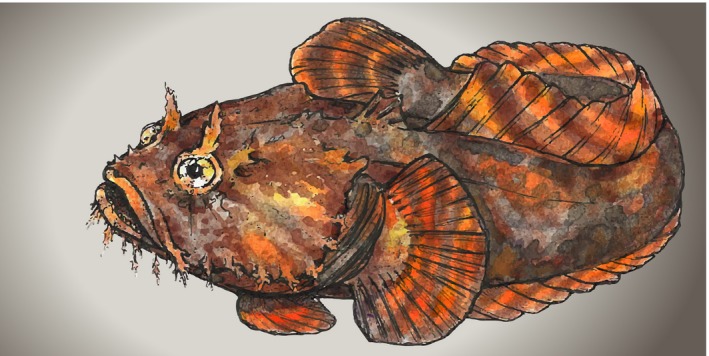
Illustration of an oyster toadfish (*Opsanus tau*). Illustration by KLZ

Oyster toadfish (*Opsanus tau*; Figure 1) represent an exemplary species in which to investigate the impact of SSD on the ontogeny of metabolically costly traits. The physiology and life history of this species has been consistently studied for over a century (Clapp, 1891; Fine, 1975; Fine, McKnight, & Blem, 1995; Fine & Waybright, 2015; Gray & Winn, 1961; Schwartz & Dutcher, 1963; Tracy, 1926), and SSD has been well documented in one unusual metabolically expensive trait: the swim bladder. The swim bladders of oyster toadfish and their close relatives (Batrachoididae) are not primarily used for buoyancy, but instead serve as highly derived sound production organs that are unusual among teleost fishes (Fine, 1975). In oyster toadfish, both males and females use these organs for grunt‐based communication (Fine & Waybright, 2015), but male swim bladders emit a specific “boat whistle”‐like call to attract mates and exhibit a nearly twofold increase in swim bladder size as a result (Fine, 1975; Fine, Burns, & Harris, 1990). The demands of this call have driven a nearly 50% increase in the size and number of fibers in sonic muscles that surround the bladder, giving rise to one of the fastest twitching muscles found in vertebrates (Fine et al., 1990). Although there is a clear metabolic cost associated with the development of these traits, the allocation of energy by male toadfish to tissue investment and the potential cost to the development of their brains or other organs remains unclear.

Here, we analyze body, brain, liver, swim bladder, heart, and gonad mass collected from a population of oyster toadfish to test whether SSD drives ontogenetic trade‐offs that support expectations of the ETH. We first quantify the allometric relationships of all organs to validate previous observations of SSD and test for differences in allometry between sexes. We then assess whether significant ontogenetic increases of organ masses with SSD are negatively correlated with the mass of other organs as anticipated by the predictions of the ETH. Results of our study provide a much needed ontogenetic investigation of SSD within the conceptual framework of the ETH and provide a critical perspective on the need for further development of theoretical expectations concerning the evolutionary relationship between sexual selection and organismal energy budgets.

## 2 MATERIALS AND METHODS

Specimens for this study were collected by research trawl across several sites in the western Delaware Bay between August and November 2016 (Greco, 2017; Figure S1). For each fish, standard and total length were recorded in millimeters (mm), and wet body mass, liver mass, heart mass, swim bladder mass, brain mass, and eviscerated body mass were recorded in milligrams (mg). Voucher photographs and otoliths were taken from each specimen and deposited in the Fish Collections of North Carolina Museum of Natural Sciences (NCSM FO 17‐28; 30; 35‐40; 43; 45‐55; 64‐75; 80‐89; 97‐98; 100‐106; 109‐116). Tests for investment trade‐offs expected under the ETH have repeatedly focused on the heart, brain, and liver as candidate organs (e.g., Aiello, 1997; Navarrete et al., 2011; Warren & Iglesias, 2012), as these are metabolically expensive and likely subjected to evolutionary trade‐offs (Konarzewski & Diamond, 1995). Since the inception of the ETH, several studies have also expanded the candidate pool of potential metabolically expensive organs subjected to trade‐offs to include gonads (e.g., Bordes et al., 2011; Liu, Zhou, & Liao, 2014; Tsuboi, Shoji, Sogabe, Ahnesjö, & Kolm, 2016). As such our study captures tissues generally considered to be among the most likely targets for metabolic trade‐offs. Thirty‐six males ranging from 91 to 262 mm and 19 females ranging from 120 to 237 mm in total length were examined (Table S1).

To quantify allometric relationships between eviscerated body mass and each organ mass for males and females, we used analysis of covariance (ANCOVA). Prior to analysis, organ and eviscerated body masses were log_10_ transformed. Although the use of a principle components analysis for extracting size as an independent measure has been used in allometric studies, this practice can drive spurious inferences of trait relationships in regression analyses (see Berner, 2011). As a result, we used eviscerated body mass as a representative of mass. Eviscerated mass was chosen over wet body mass as the stomach contents of individual fishes varied considerably, from no prey to individuals containing copious amounts of shellfish or conspecifics almost a quarter of their body size. A linear model was fit with tissue mass as the dependent variable and eviscerated body mass, sex, and sex*eviscerated body mass as independent variables. Body mass was treated as a covariate, and sex difference was assessed by determining whether separate regression lines fit the data better than a single regression line. Sex difference was tested using an *F* test with two degrees of freedom (*df*) in the numerator, one *df* for a sex difference in slope, and the other *df* for a sex difference in intercept. An alpha level of 0.05 was used to infer a sex difference in body mass allometry for each organ. When an allometric difference between sexes was inferred, the result for a test for difference in slope was reported.

To evaluate ETH for male swim bladder as a hypothetically expensive tissue, we estimated partial correlation coefficients between swim bladder and each of four candidate tissues (gonad, heart, brain, and liver). As applied here, partial correlation measures linear association between swim bladder and a candidate tissue while controlling for the influence of body mass (see Supporting Information). When controlling for a single variable, in this case body mass, the partial correlation coefficient can be obtained from three correlation coefficients, swim bladder—body mass, target organ—body mass, and swim bladder—target organ (see Supporting Information). Again, all tissue masses were log_10 _transformed prior to computing correlations, and 95% confidence intervals for partial correlation coefficients were based on a *t* distribution with *n *− 3 degrees of freedom. A negative partial correlation coefficient for a target organ supports an ETH interpretation for swim bladder. Results obtained through partial correlation were additionally compared to multivariate and univariate regression (see Supporting Information). Additionally, to account for a potential confounding effect of sonic muscles in the fish trunk following evisceration, similar correlation analyses were repeated using standard length (mm) as a measure of body size (see Supporting Information).

## 3 RESULTS

**Figure 2:**
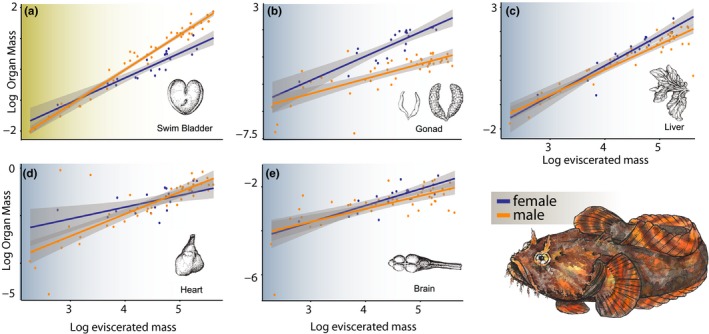
Patterns of sexual size dimorphism (SSD) in oyster toadfish for (a) swim bladder, (b) gonad, (c) liver, (d) heart, and (e) brain masses. Females are depicted in blue, and males are depicted in orange. Light shading of the plot indicates significant evidence for SSD based on an ANCOVA

We found significant allometric differences between males and females for swim bladder mass (*F*_2,56_ = 13.38, *p* < .0001), gonad mass (*F*_2,55_ = 42.02, *p* < .0001), and liver mass (*F*_2,56_ = 12.13, *p* < .0001). No significant difference between sexes was found for heart mass (*F*_2,56_ = 2.902, *p* = .063) or brain mass (*F*_2,56_ = 1.924, *p* = .155). ANCOVA results supported a sex difference in slope for the swim bladder and body mass relationship (*F*_1,56_ = 20.06, *p* < .001), providing strong evidence for a difference in allometric growth of the swim bladder between males and females. Visualizations of the allometric trajectory for swim bladder mass show large males possess larger swim bladders than females of comparable body mass (Figure 2). Females appear to diverge from males in the slope of the regression of gonad on body mass (Figure 2); however, the one *df* test for a sex difference in slope did not achieve statistical significance (*F*_1,56_ = 3.762, *p* = .058). Likewise, no strong support was found for an allometric difference between sexes in liver mass (*F*_1,56_ = 0.978, *p* = .327; Figure 2).

**Figure 3:**
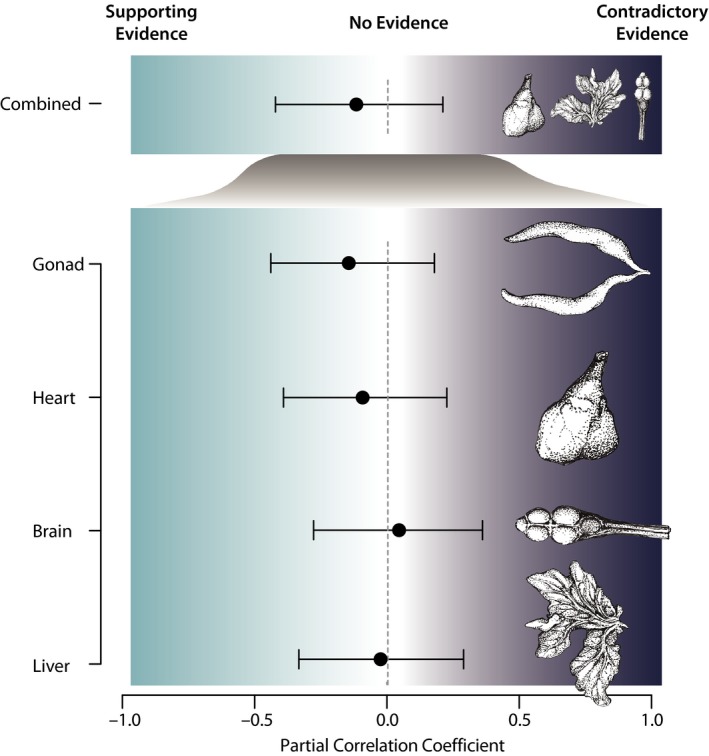
Partial regression coefficient estimates for linear relationships between mass of swim bladder and other putative metabolically expensive tissues. A 95% confidence interval for a negative correlation coefficient that excludes zero would support the expectation of the expensive tissue hypothesis (ETH) (light shading), while a confidence interval for a positive coefficient that excludes zero would provide contrary evidence (dark shading). Partial correlation intervals that include 0 provide no evidence for or against the expectations of the ETH

Point estimates and associated 95% confidence intervals for partial correlation coefficients between swim bladder and four organs (gonad, heart, brain, and liver) are plotted in Figure 3. Point estimates for three partial correlation coefficients are negative and one is positive. However, all confidence intervals include zero (Figure 3), thereby providing no strong evidence for a negative linear association between swim bladder and any of the four internal organs individually examined. The sum of heart, brain, and liver mass was also evaluated for a negative association with swim bladder and exhibited the same result as individual organs. Collectively, all of our results provide no support for a negative correlation between swim bladder mass and the mass of any other candidate organ and thereby do not support the expectations of the ETH.

## 4 DISCUSSION

Swim bladder SSD has been well documented in oyster toadfish (Fine, 1975; Fine et al., 1990). Fine (1975) proposed that the size differences in the toadfish swim bladder are the result of different ontogenetic growth trajectories between males and females. Given little variation between the ontogenies of individual males and females, we would expect similar ontogenetic (individual) and static (population) allometric slopes. Our findings support this hypothesis, showing that an increase in static allometric slope in males relative to females underlies the sex‐related difference in swim bladder size (see Figure 2). For species characterized by SSD, including those with highly exaggerated traits such as weapons and ornaments, positive changes in static allometric slope, where larger individuals have disproportionately larger traits, are far less common than negative changes or changes in intercept (isometry; reviewed in Bonduriansky, 2007). The lack of evidence for a tight coupling between positive change in allometry and SSD is consistent with the hypothesis that allometric slopes are not very evolvable below the species level (Voje, Hansen, Egset, Bolstad, & Pélabon, 2014). In species where changes in ontogenetic or static allometric slope have been found, two explanations are commonly invoked. First, changes in slope driven by shifts in environment or resource utilization can promote rapid phenotypic divergence (e.g., Collins, Dornburg, Flores, Dombrowski, & Lewbart, 2016; Ruehl, Shervette, & Dewitt, 2011; Wund, Valena, Wood, & Baker, 2012). This explanation seems unlikely for oyster toadfish as swim bladders of both sexes are not used for buoyancy, but rather communication, and males and females overlap in habitat and diet (Wilson 1982). Alternatively, sexual selection theory predicts that strong selection on trait exaggeration with a low overall cost to the organism can promote allometric slope changes (Bonduriansky & Day, 2003). This hypothesis is appealing; however, our study does not offer a quantification of energetic costs associated with the swim bladder. Further, we do not believe this selection hypothesis offers a complete explanation. Instead, we propose that the changes in static allometric slope we report here may have deep evolutionary origins.

Other species of Batrachoidiformes, such as members of the genus Porichthys (Midshipman), have been found to be sexually dimorphic in swim bladder size (Mohr et al., 2017). Given that Porichthys and Opsanus share common ancestry at least 30 million years ago (Near et al., 2013), it is likely that the changes in ontogenetic slope found in our study may represent an axis of Batrachoidiform diversification that vastly predates the origin of *Opsanus tau*. Given these deep origins, the energetic costs associated with this organ may have long been integrated into the ontogeny of these organisms. Consequently, our study may represent a small component of a macroevolutionary shift in evolutionary allometry. Future work placing the evolutionary allometry of Batrachoidiform swim bladders into a phylogenetic framework and assessing the costs and developmental mechanisms of ontogenetic change between sexes of multiple species represents exciting frontiers that are necessary to evaluate the origin of this unusual organ and its related musculature.

Despite finding clear evidence of a significant change in the allometric slope of swim bladder growth between toadfish sexes, our analyses did not recover any evidence of a correlated trade‐off in the mass of another organ during ontogeny (Figure 2). Male toadfish swim bladder mass has previously been found to be highly correlated with both sonic muscle size and the number of fibers (Fine, 1975), making swim bladder mass a good proxy for the heavy energetic cost associated with the development of the male toadfish acoustic repertoire. It is certainly possible that a trade‐off between a tissue or life history trait and swim bladder mass may exist and was simply not examined here. Given that we find no significant negative change in brain, liver, or heart allometric trajectories, our study adds to the growing number of studies that have failed to recover support for the ETH in traits where an expectation of positive evidence is reasonable (reviewed in Warren & Iglesias, 2012).

The decoupling of ontogenetic changes between the swim bladder and other organs suggested by our results raises the possibility that the ontogeny of the swim bladder and related musculature may represent a morphological module. Modularity, the degree of separation of one axis of phenotype from other organismal parts, is a fundamental principle of biological organization (Esteve‐Altava, 2017). However, the expectations of the ETH suggest that the modular organization of biological forms can impact the development of the brain as a consequence of resource allocation constraints. We found no clear relationship between brain mass and the mass of any other organ. This raises the question: To what extent should we expect increased investment of one module to directly negatively impact the ontogenetic trajectory of another?

Studies of resource allocation trade‐offs have suggested tissue proximity to be a potential predictor of changes in investment (Emlen, 2001). Although organisms must use a finite energy budget to accumulate body mass, the ubiquity of modularity in organismal systems ranging from mammals to fishes (Esteve‐Altava, 2017; Larouche, Cloutier, & Zelditch, 2015) without obvious trade‐offs between adjacent tissues (reviewed in Warren & Iglesias, 2012) suggests that simple economic predictions between morphological modules may not have much explanatory power for understanding the evolution of most ontogenetic pathways without a more detailed perspective of lineage‐specific energy budgets and life history. While investment trade‐offs between morphological modules have provided evidence for the expectations of the ETH in a few animal lineages (Emlen, 2001; Liao et al., 2016; Moczek & Nijhout, 2004), the large number of studies that have failed to recover support in other lineages suggests that the broad expectations of the ETH are far from a universal rule (reviewed in Warren & Iglesias, 2012).

While increases in the energetic cost of one morphological module do not often lead to negatively correlated changes in another, this does not preclude the possibility that some modules are in fact faced with a possible deficit in energy as sexual selection emphasizes trait investment (Moczek & Nijhout, 2004). However, without a detailed understanding of the ecology and energy requirements of a species, it is not clear to what extent organisms can offset deficits through changes in behavior or feeding ecology. Such subtle changes may in part explain the strong evidence for the ETH in experimental laboratory settings with tight controls (Kotrschal et al., 2013, 2015, 2016), despite limited support from wild populations such as the toadfishes in this study. Further, an organism is comprised of a suite of morphological modules that collectively use an energy budget to invest mass into their respective structures. Numerous subtle increases or decreases to energy consumption across any number of modules can therefore offset the cost of strong sexual selection to a specific module. Such a readjustment of energy budgets is reminiscent of many‐to‐one mapping of form to function, where numerous phenotypic solutions give rise to the same functional properties of a trait (Wainwright, Alfaro, Bolnick, & Hulsey, 2005). As such, the repeated lack of evidence for the ETH may not result from a lack of trade‐offs, but rather from subtle and complex adjustments of ontogenetic investment between numerous morphological modules across an entire organism that cannot be detected through approaches based on tissue mass alone. Future studies assessing the behavior, feeding ecology, and energy costs of different morphological modules will be needed to determine whether any of these hypotheses explain the lack of trade‐offs between the male toadfish swim bladder and other organs.

## 5 Conclusion

Determining the impact of energetic trade‐offs in the ontogenetic pathways that give rise to the diversity of phenotypes we observe today is fundamental to evolutionary biology. While the ETH holds promise of a general evolutionary principle, evidence for direct trade‐offs between the brain and other metabolically expensive organs has been limited to few clades. In contrast, numerous studies, including this study, have reported negative evidence within and among a broad spectrum of clades. While a one‐to‐one mapping of brain investment increase to trait reduction does appear to exist in some species (Kotrschal et al., 2013; Tsuboi et al., 2015, 2016), these examples are few in number. Refinement of the ETH as well as the formulation of new metabolic investment hypotheses is warranted and needed to broaden our perspective on energetic trade‐offs. Such hypotheses are critical if we are to develop new insights into the role of sexual selection in shaping other aspects of organismal form.

## CONFLICT OF INTEREST

None declared.

## AUTHOR CONTRIBUTIONS

A.D. and R.W. conceived of the study. R.W. collected samples. A.D., K.L.Z., A.L., G.H., and L.L. collected data. R.M., A.D., and D.W. performed analyses. A.D., D.W., K.L.Z., R.M., T.L.I., A.L., and R.W. wrote the initial manuscript. All other authors contributed to the subsequent writing and development of the manuscript. All data have been archived on Zenodo: (DOI pending formal acceptance).
