# Supplementary figures and images for: Testing ontogenetic patterns of sexual size dimorphism against expectations of the expensive tissue hypothesis, an intraspecific example using oyster toadfish (Opsanus tau)
Source: Ecol Evol. 2018 Mar 2;8(7):3609–16. doi: 10.1002/ece3.3835 (PMC5901164; doi:10.1002/ece3.3835)

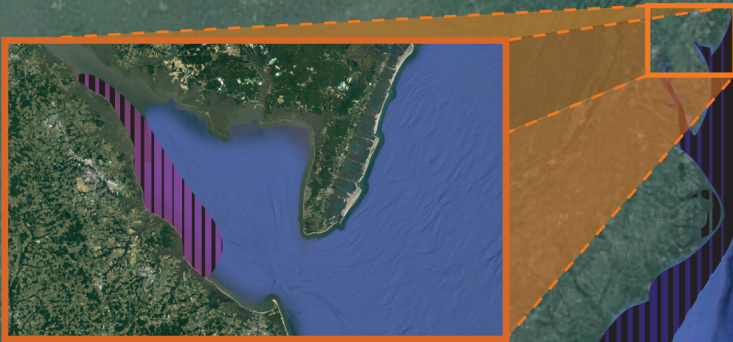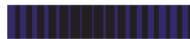

Coastal range

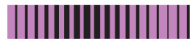

Sampled Area

Supplement: Supplementary file 1 [file ECE3-8-3609-s001.pdf]

A.

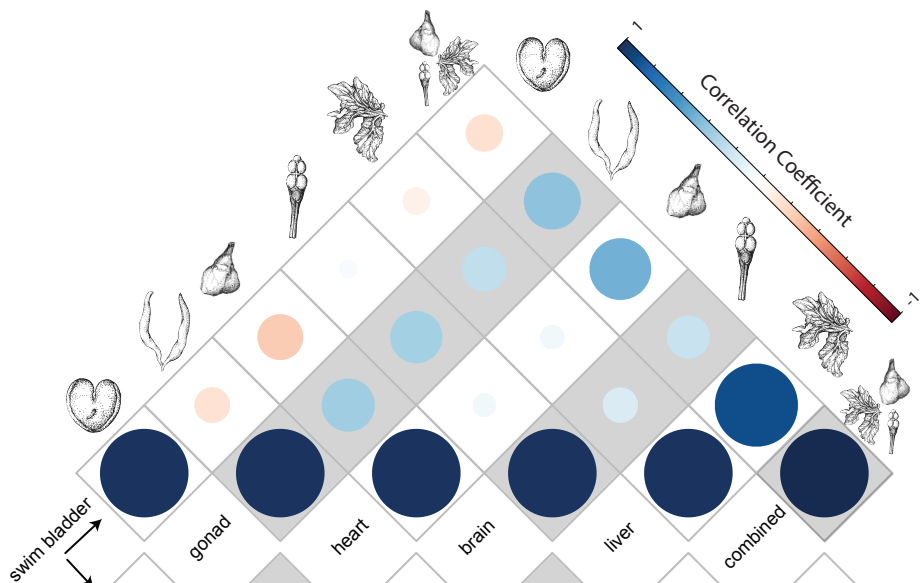

B.

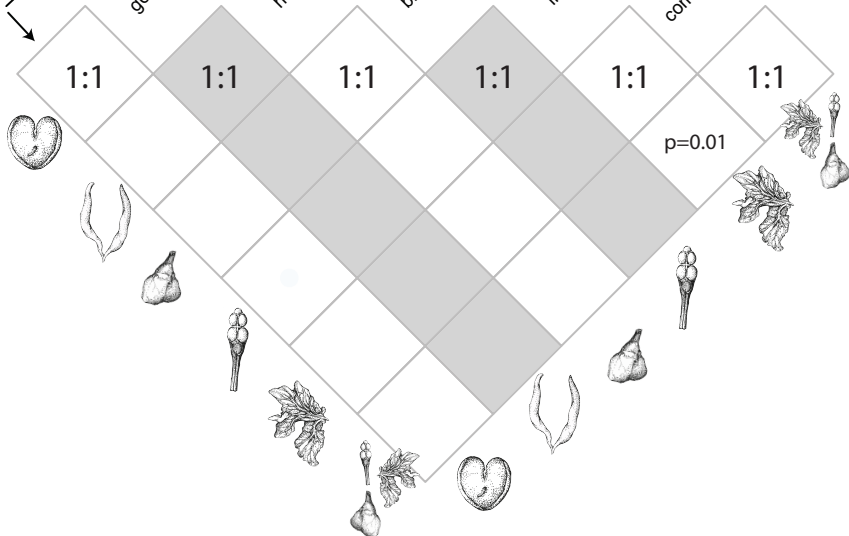

Supplement: Supplementary file 2 [file ECE3-8-3609-s002.pdf]

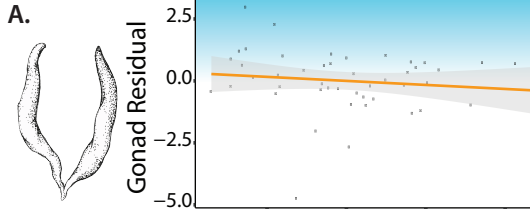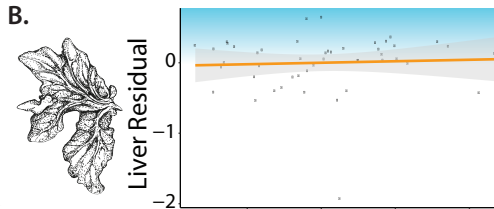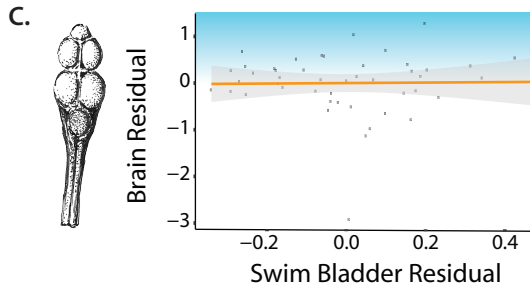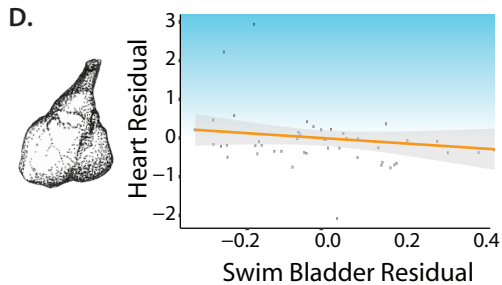

Supplement: Supplementary file 4 [file ECE3-8-3609-s004.pdf]

A.

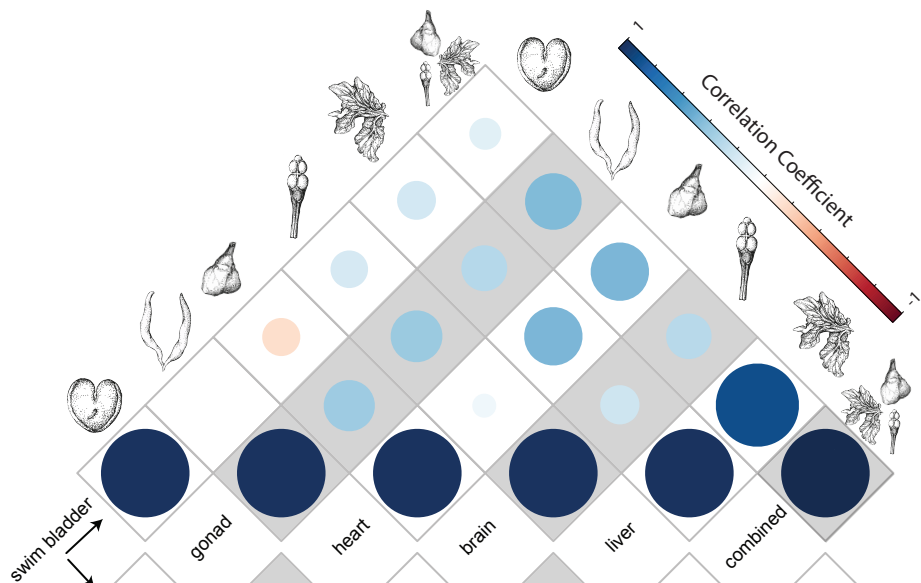

B.

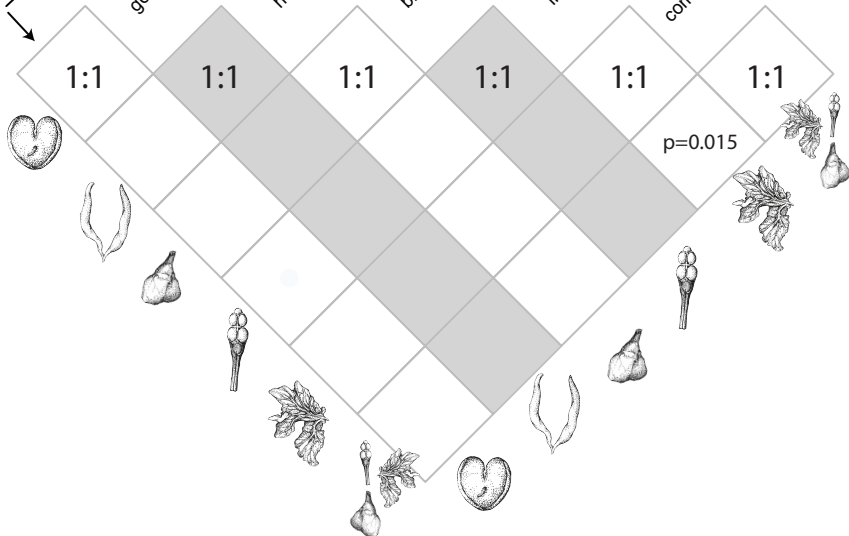

Supplement: Supplementary file 5 [file ECE3-8-3609-s005.pdf]
