# Supplementary material for: Testing ontogenetic patterns of sexual size dimorphism against expectations of the expensive tissue hypothesis, an intraspecific example using oyster toadfish (Opsanus tau)
Source: Ecol Evol. 2018 Mar 2;8(7):3609–16. doi: 10.1002/ece3.3835 (PMC5901164; doi:10.1002/ece3.3835)
Supplement: Supplementary file 3 [file ECE3-8-3609-s003.pdf]

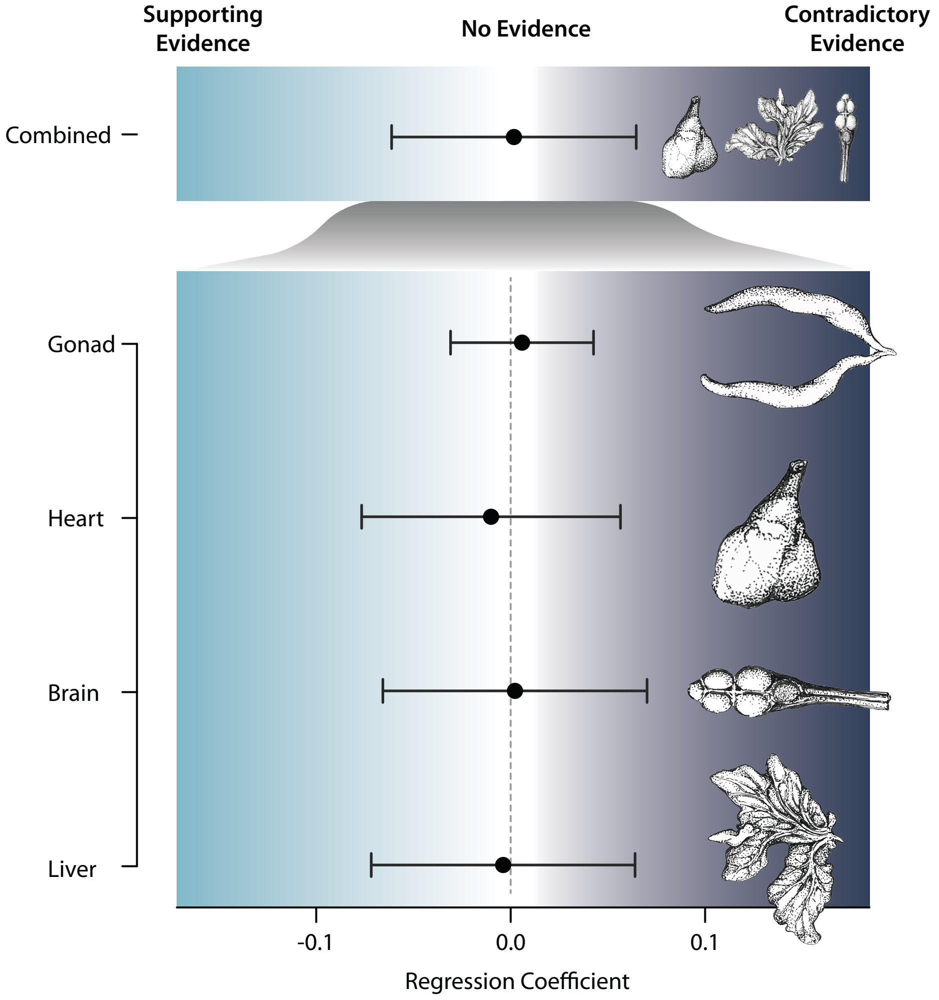

Supporting Evidence
No Evidence
Contradictory Evidence
Combined
Gonad
Heart
Brain
Liver
-0.1
0.0
0.1
Regression Coefficient
