# Supplementary material for: Testing ontogenetic patterns of sexual size dimorphism against expectations of the expensive tissue hypothesis, an intraspecific example using oyster toadfish (Opsanus tau)
Source: Ecol Evol. 2018 Mar 2;8(7):3609–16. doi: 10.1002/ece3.3835 (PMC5901164; doi:10.1002/ece3.3835)
Supplement: Supplementary file 6 [file ECE3-8-3609-s006.docx]

*Supplemental materials*

***Methods***

*Partial correlation*

Partial correlation measures linear association between two variables while controlling for one or more other variables, and has been suggested as a useful measure of effect size in biological studies (Nakagawa and Cuthill 2007). Correlation is an intuitively attractive measure because it is unit-less, lies in a closed interval [-1,1] and is symmetric in x and y. The partial correlation coefficient derives from a partial regression coefficient that, in this application, estimates the slope for tissue effect on swim bladder adjusted for body mass in a multiple regression model. The partial regression coefficient can be standardized resulting in a standardized partial regression coefficient, which is closely related to the partial correlation coefficient. In this study, there is a single covariate, body mass, to be controlled in assessing the linear relationship between swim bladder mass and a candidate tissue mass. The partial correlation is numerically equivalent to the correlation between residuals from separately regressing swim bladder on body mass and candidate tissue on body mass. Residuals from the same individual are treated as paired data in computing Pearson’s product moment correlation. Alternatively, when there is a single covariate to be controlled the partial correlation coefficient can be computed explicitly as a function of three zero-order correlation coefficients using the following expression

$$r_{xy.z}=(r_{\mathrm{xy}} - r_{\mathrm{xz}} r_{\mathrm{yz}})/(\surd(1-r_{\mathrm{xz}}^{2})(1-r_{\mathrm{yz}}^{2}))$$

Here x represents swim bladder, y represents candidate tissue and z represents body mass.

*Multivariate and Univariate Regression*

In addition to the above approaches, estimates of the correlation between swim bladder residuals and residuals of each of the other tissues (i.e. brain, liver, gonad and heart) after accounting for body mass were obtained by fitting a multivariate linear regression model. In this model, the dependent variable is a vector of log-transformed organ masses, [swim bladder, brain, liver, gonad, heart]^T^, and the independent variable is a log-transformed body mass. Partial correlation coefficients between elements of the multivariate dependent variable, which include partial correlations between swim bladder and each of the other tissues, can be obtained by computing correlation coefficients from the variance-covariance matrix of residuals from this model. When swim bladder and other tissue masses contain no missing values, correlations obtained from this approach are the same as those obtained from the above described procedures. Analyses were also repeated using univariate regression as this approach is commonly used for studies of this type (e.g., Berner 2011). All analyses were conducted in R.

***Results***

*Multivariate and Univariate Regression*

Results of a multivariate regression correspond to results obtained through partial correlation analysis. We recover a mixture of small positive and negative partial correlation coefficients (Supplemental Figure 2), however, no significant effect was detected between swim bladder mass and other masses from other candidate organs. Likewise, univariate regression yielded regression coefficients whose estimates overlap with zero (Supplemental Figure 3), and identical correlations to the multivariate results when data was standardized to include no missing values. Plots of the residual variation of organ mass on swim bladder mass following a regression on body mass demonstrating the lack of trend expected when no strong relationship is present (Supplemental Figure 4).

**Supplemental Tables**

|  | Eviscerated Mass (g) | Gonad Mass (g) | Liver Mass (g) | Brain Mass (g) | Swim Bladder Mass (g) | Heart Mass (g) | Total Length (mm) | Standard length (mm) |
| --- | --- | --- | --- | --- | --- | --- | --- | --- |
|  | Mean/SD/n | Mean/SD/n | Mean/SD/n | Mean/SD/n | Mean/SD/n | Mean/SD/n | Mean/SD/n | Mean/SD/n |
| Male | 112.10/87.22/41 | 0.47/0.46/40 | 3.35/2.96/41 | 0.08/0.05/39 | 2.27/1.94/41 | 0.31/0.26/41 | 182.96/54.72/46 | 157.25/48.64/46 |
| Female | 98.69/46.92/19 | 5.83/5.02/19 | 3.89/1.97/19 | 0.10/0.05/19 | 1.18/0.51/19 | 0.26/0.13/19 | 180.82/29.49/23 | 154.78/24.75/23 |

Supplemental Table 1: Summary statistics of morphological data. SD=Standard deviation, n=sample size, g=grams.

**Supplemental Figure Legends**

Supplemental Figure 1. Partial Geographical range of oyster toadfish in Eastern North America and location of area sampled (Google Maps, 2017).

Supplemental Figure 2. Summary of multivariate regression. A. Correlation coefficients estimated for all comparisons. Circle sizes and color indicate strength of correlation indicated in the legend. Strong negative (red) correlations would support the expectations of the ETH. B. Significance tests of each correlation. Diagonal of matrix indicated by 1:1. Only p values less 0.05 are shown.

Supplemental Figure 3. Regression coefficients for relationship between mass of swim bladder and other putative metabolically expensive tissues. Negative regression coefficient estimates with 95% confidence intervals that exclude zero would support the expectations of the ETH (light shading), while positive regression coefficient estimates with 95% confidence coefficient intervals that exclude zero would provide contrary evidence (dark shading). Confidence intervals that overlap with 0 provide no evidence for or against the expectations of the ETH.

Supplemental Figure 4. Residuals from log-log regression of organ mass versus body mass plotted against residuals from log-log regression of swim bladder mass versus body mass. Colored gradients in each plot depict the area above the origin.

Supplemental Figure 5. A. Correlation coefficients estimated for all comparisons using total length as a measure of body size. Circle sizes and color indicate strength of correlation indicated in the legend. Strong negative (red) correlations would support the expectations of the ETH. B. Significance tests of each correlation. Diagonal of matrix indicated by 1:1. Only p values less 0.05 are shown.
